# Using Chemical Ecology to Enhance Weed Biological Control

**DOI:** 10.3390/insects12080695

**Published:** 2021-08-03

**Authors:** Alexander M. Gaffke, Hans T. Alborn, Tom L. Dudley, Dan W. Bean

**Affiliations:** 1 Center for Medical, Agricultural, and Veterinary Entomology, Agricultural Research Service, United States Department of Agriculture, Gainesville, FL 32608, USA; agaffke@agcenter.lsu.edu (A.M.G.); hans.alborn@usda.gov (H.T.A.); 2 Department of Entomology, Louisiana State University Agricultural Center, Baton Rouge, LA 70803, USA; 3 Marine Science Institute, University of California, Santa Barbara, CA 93106, USA; tdudley@msi.ucsb.edu; 4 Colorado Department of Agriculture, 750 37.8 Rd, Palisade, CO 81526, USA

**Keywords:** semiochemical, aggregation pheromone, biological control implementation, GC-EAD

## Abstract

**Simple Summary:**

Signaling chemicals produced by one organism that bring about a behavioral response in a recipient organism are known as semiochemicals, with pheromones being a well-known example. Semiochemicals have been widely used to monitor and control insect pests in agricultural and forestry settings, but they have not been widely used in weed biological control. Here, we list the few examples of semiochemical use in the practice of classical weed biological control, where a natural enemy (biocontrol agent) from the native range of the plant is introduced into the new invaded range. Uses of semiochemicals include monitoring of biocontrol agents (sex pheromones), keeping biocontrol agents together long enough for them to become well established (aggregation pheromones) and repelling agents from areas where they may be unwanted (host or non-host plant volatile organic deterrents). We make the case that given the vast potential of biological control in suppressing invasive plants it is well worth developing and utilizing semiochemicals to enhance biocontrol programs.

**Abstract:**

In agricultural systems, chemical ecology and the use of semiochemicals have become critical components of integrated pest management. The categories of semiochemicals that have been used include sex pheromones, aggregation pheromones, and plant volatile compounds used as attractants as well as repellents. In contrast, semiochemicals are rarely utilized for management of insects used in weed biological control. Here, we advocate for the benefit of chemical ecology principles in the implementation of weed biocontrol by describing successful utilization of semiochemicals for release, monitoring and manipulation of weed biocontrol agent populations. The potential for more widespread adoption and successful implementation of semiochemicals justifies multidisciplinary collaborations and increased research on how semiochemicals and chemical ecology can enhance weed biocontrol programs.

## 1. Introduction

Invasive plant species are a global problem, impacting and threatening the sustainability of agriculture and ecosystems [1,2]. Classical weed biocontrol, a management program that provides safe and effective options for control of invasive plant species [3], involves the introduction and establishment of host specific coevolved herbivores from the native range of the invasive plant, with the aim of facilitating permanent suppression of the plant. Since the herbivore and plant host are co-evolved organisms in the regions of origin, there is an increased likelihood that the herbivore will be highly host specific. In addition, a candidate agent goes through significant prerelease evaluation of host specificity and potential impact to the target weed as well as to non-target non-invasive domestic plants [3,4]. Host specificity involves a complex set of ecological and physiological relationships in part mediated by semiochemicals, which are chemicals produced by one organism that elicit a behavioral response (mating, food finding, predator avoidance, etc.) in the recipient organisms. The study of semiochemicals and their impact on behavior is known as chemical ecology, and the principles of chemical ecology are instructive in defining semiochemically mediated interactions between a host plant and co-evolved herbivores, such as those used in weed biocontrol.

The field of chemical ecology has been widely adopted in integrated pest management and numerous pest control programs have succeeded due to the deployment of semiochemicals [5,6]. However, the field of weed biocontrol has been slow to adopt principles of chemical ecology even though it has been long recognized as critical to understanding host range [7] and the behavioral and physiological interactions between and among host plants and biocontrol agents could be better understood and manipulated using methods developed by chemical ecologists. The incorporation of chemical ecology into host range testing, selection of agents, and post release interaction with the target plant has been reviewed by Wheeler and Schaffner [8]. The review focused primarily on the chemistry of host specificity, evolutionary changes in plant chemistry, herbivore induced compounds, variation in compounds, and sequestration of compounds. The integration of these research approaches into host range and efficacy testing will almost certainly increase our ability to predict host ranges and increase our confidence in the selection of safe and effective biocontrol agents. Once an agent has been determined to be safe, and has been approved for open field release, there are further ways that chemical ecology could be used in the implementation of weed biocontrol to increase agent impact on the target plant and enable better integration of weed biocontrol into invasive plant management programs. 

Our goals for this report are to describe the few systems where semiochemicals have been used to enhance weed biocontrol (see Table 1), and to encourage biocontrol practitioners to incorporate chemical ecology into weed biocontrol implementation. After outlining methods of semiochemical discovery with examples from weed biocontrol, we discuss three points in the implementation of weed biocontrol where semiochemicals, and the principles of chemical ecology, can be useful. First is in establishment of weed biocontrol agents using aggregation pheromones. Second is in monitoring establishment and range expansion of weed biocontrol agents, using pheromones, and finally we discuss the use of pheromones and plant-produced semiochemicals to manipulate established agent populations in the field. We also encourage biocontrol practitioners to work with chemical ecologists to develop novel approaches to enhance the practice of weed biocontrol. Cooperation between resource managers, field biologists and chemical ecologists could produce a rich stream of new techniques to benefit weed biocontrol, while also increasing basic knowledge of the chemical ecology of herbivorous insects.

## 2. Discovery and Development of Semiochemicals for Weed Biocontrol 

**Figure 1:**
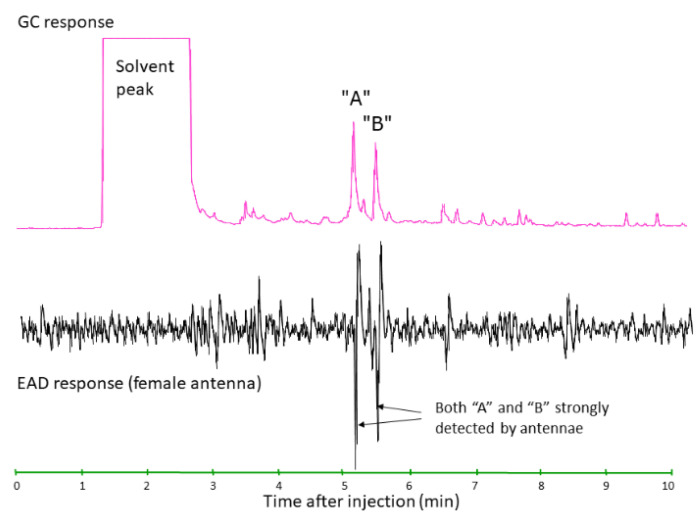
An example of the output of gas chromatographic-electroantennagraphic detection (GC-EAD) of volatiles from feeding *D. carinulata* adult males [15]. This shows the electrophysiological response of an isolated insect antenna (lower line, in black) to GC fractionated volatile compounds collected from feeding adults of *D. carinulata* (upper line, in pink), enabling researchers to isolate compounds that are antennally active (e.g., the *D. carinulata* pheromone blend, compounds A&B, (2E,4Z)-2,4-heptadienal and (2E,4Z)-2,4- heptadien-1-ol, respectively, which were fractionated from the volatiles mixture). Figure reproduced with permission from [15], copyright 2005 SpringerNature.

The typical pathway for development of semiochemicals as weed management tools begins with field-based observations, such as the observation of aggregation of conspecifics, long-range mate finding, or attraction to host plants during dispersal. Observations can then lead to hypotheses regarding the role of semiochemicals in modulating herbivore behavior. The next steps occur in specialized laboratory settings where behaviorally active volatile compounds are captured from an air stream passed over living insects or plants (or insects feeding on plants) in settings designed to mimic field conditions., The mix of collected volatile compounds is fractionated using gas chromatography (GC), and antennally active compounds are identified from the fractionated compounds using electroantennagraphy detection (EAD) to produce parallel plots (GC-EAD) [20], which are useful in identification of volatile compounds that the insect may perceive in the field (Figure 1). The chemical structures of fractionated compounds with antennal activity are determined using an array of physical chemical techniques (see [21] for examples). Behavioral assays can then be used to answer the question “Do these compounds elicit measurable behavioral responses from the insect?” 

There are a variety of behavioral assays with choice tests being typical for measuring behavioral responses to semiochemicals. Choice tests are often conducted in olfactometers, which measure chemotaxis in an arena where the insect is subjected to air streams with and without the test compound or compounds (e.g., [17]). The locomotory response of the insect in an olfactometer is then typically broadly categorized as being either attractive, neutral, or repellent to the insect. An attractant versus repellent causes the mover to make oriented movements toward versus away from the source of stimulation [22]. Olfactometer tests provide a measure of biological activity which is followed by field-testing to demonstrate behavioral activity under conditions encountered in natural settings [15,16,19]. The final step in deployment is formulation of the compound into a delivery system that will allow release in a way that mimics a natural source (e.g., [23]). The expertise and equipment required makes semiochemical development challenging, but we believe the benefits substantially outweigh the costs. All weeds targeted for biocontrol have, by definition, major economic and environmental impacts coupled with other properties that make them costly, usually environmentally damaging, and difficult to control using other methods. Enhancing the efficacy of weed biocontrol agents is a valuable investment.

## 3. Sex Pheromones

Sex pheromones that attract potential mates, sometimes from great distances, have been extensively used in pest management for monitoring or mass trapping of harmful insects and for disruption of mating behavior, with hundreds of examples [24]. Sex pheromones are typically emitted by reproductive females and are attractive to males in settings where insect densities are low [25]. Sex pheromones are common in the Lepidoptera, where short-lived adults may be present in low densities requiring pheromonal signals to improve the chances of encountering potential mates [25,26]. There are only four examples of this application for monitoring moths used as classical biocontrol agents [27]. The first examples were from the gorse, *Ulex europaeus* L., biological control program in New Zealand where two moth species, the gorse pod moth, *Cydia succedana* (Denis & Schiffermüller) (Lepidoptera: Torticidae) and the gorse soft shoot moth, *Agonopterix umbellana* (Fabricius) (Lepidoptera: Depressariidae) were monitored with sex attractants screened from known pheromonal components of congeneric species [9,10]. The compounds were attractive but not fully characterized as pheromones, and so were placed in the broader category of sex attractants. Sex attractants were then deployed for monitoring two moths used against invasive *Rubus* spp in Hawaii [11]. A pheromone blend was developed to monitor the cactus moth *Cactoblastis cactorum* (Berg) (Lepidoptera: Pyralidae) a biocontrol agent accidentally introduced outside of its intended range [14]. 

Sex pheromones for two biocontrol agents were identified but never used to monitor populatons in the field. A pheromone blend was identified for *Tyta luctuosa* (Denis & Schiffermüller) (Lepidoptera: Noctuidae), a biological control agent for field bindweed, *Convolvulus arvensis* L. [12]. The blend was never available for field use, probably because *T. luctuosa* was not known to be established in the field until several years after the study was completed (D. Bean, personal observation). A pheromone blend was also identified for the root mining moth *Agapeta zoegana* L. (Lepidoptera, Tortricidae) a biocontrol agent for knapweeds (*Centaurea* L. spp.) in North America [13]. Although one component was very effective in trapping male moths in the native range of *A. zoegana* (Hungary) it was never deployed in North America probably because work on the sex pheromone was completed prior to establishment of *A. zoegana* in western North America.

## 4. Aggregation Pheromones

Unlike sex pheromones, aggregation pheromones are attractive to both sexes and are usually emitted by males [28]. They bring about a localized increase in density of reproductive adults, often resulting in feeding damage, oviposition and large populations of larvae in the subsequent generation, all of which impose substantial stress on the host plant. Aggregation pheromones are well represented in the herbivorous Coleoptera, especially in the families Chrysomelidae and Curculionidae [28], which include many of the most successful weed biocontrol agents [29]. Despite this, there are only two weed biocontrol agents in which an aggregation pheromone or pheromone blend has been fully characterized, and one genus where aggregation pheromones are likely, given preliminary results, as described below. It is reasonable to hypothesize a role for aggregation pheromones in the biology of many more agents that are known to achieve localized high densities of conspecific adults on the host plant. 

Adults of the purple loosestrife beetles *Galerucella calmariensis* L. and *G. pusilla* Duftschmidt (Coleoptera: Chrysomelidae) locate the host plant purple loosestrife (*Lythrum salicaria* L.), aggregate, mate and oviposit and the resulting larvae can defoliate the entire plant [30]. Studies revealed not only the effective dispersal distance for *Galerucella* but also a role for conspecifics in attracting dispersing insects to previously colonized patches of purple loosestrife [31], presumably increasing the likelihood of successful establishment by increasing population density [32]. Those studies provided behavioral observations supporting the existence of aggregation pheromones in *Galerucella* while later studies led to the identified a novel dimethylfuran lactone as an aggregation pheromone in *Galerucella* spp. [18]. Further work showed how the aggregation pheromone is part of the biology of *Galerucella*, enabling host plant location and aggregation in purple loosestrife beetles [19]. Males were postulated to initiate dispersal, locating plants based upon host chemical cues. When males discover acceptable host plants they emit the aggregation pheromone, calling in conspecifics to increase local population densities capable of initiating defoliation while reducing the risk of local extirpation at low population densities (i.e., overcoming potential Allee effects in which individual fitness decreases at low population densities) [19,32]. 

**Figure 2:**
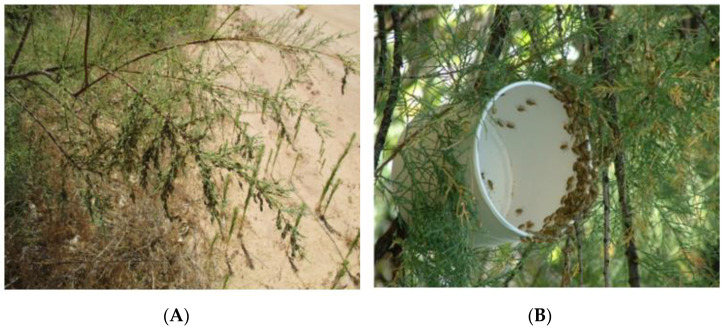
(**A**) Aggregation of *Diorhabda carinulata* on *Tamarix*. (**B**) A field release of *D. carinulata* from a cardboard container, typical for insect biocontrol releases.

**Figure 3:**
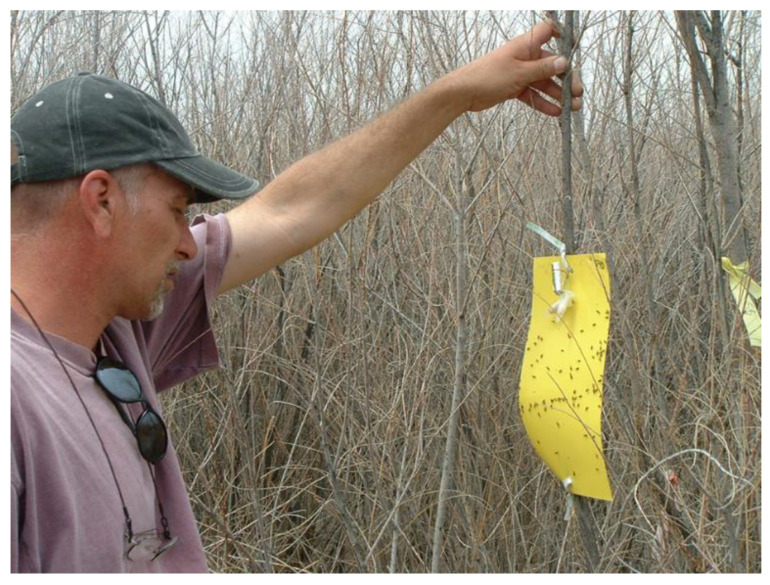
Yellow sticky cards baited with the antennally active compounds, *D. carinulata* pheromone blend + green leaf volatiles, as part of a field trial conducted in western Nevada on 5 April 2004. Attraction of adult beetles to the cards was the measure of semiochemical activity of the volatile compounds. Allard Cossé, chemical ecologist with the USDA ARS, is here seen checking the trap as part of a cooperative effort between USDA ARS chemists and biocontrol practitioners [15,16].

The northern tamarisk beetle, *Diorhabda carinulata* Desbrochers (Coleoptera: Chrysomelidae) was introduced into the field in North America in 2001 for control of the invasive riparian shrub tamarisk (*Tamarix* L. spp., hereafter indicated as *Tamarix*). By 2003, large areas of *Tamarix* had been defoliated and it had been noted that beetles displayed aggregation behaviors which resulted in high densities of adults capable of leaving sufficient offspring to defoliate entire plants (Figure 2). Antennally active compounds were found in volatiles collected from feeding, reproductive male beetles and field-testing verified pheromonal activity in two of these [15]. This work was part of a cooperative effort in which biocontrol practitioners located field sites and provided logistic support while chemical ecologists from the United States Department of Agriculture, Agricultural Research Service (USDA ARS) developed semiochemicals for testing in the field using baited yellow sticky traps ([15], Figure 3). A male-produced blend of two pheromone components, (2E,4Z)-2,4-heptadienal and (2E,4Z)-2,4- heptadien-1-ol, is attractive to reproductive male and female beetles, leading to formation of reproductive aggregations in this species [15] increasing population density and overall impact on the target plant.

Eight male-produced volatile compounds were described in three species of *Aphthona* Chevrolat spp., flea beetles (Chrysomelidae: Alticinae) used for biocontrol of leafy spurge [21], and separate trials done with *A. nigriscutus* Foudras showed that conspecifics were attracted to feeding males in a behavioral assay [33]. In combination, these studies provided strong evidence for a male produced aggregation pheromone in leafy spurge flea beetles, fitting well with observed behavior in which adult flea beetles attain high densities, in patchy distributions [34].

The first fully characterized male-produced aggregation pheromone identified from the Chrysomelidae was isolated from a serious agricultural pest, the Colorado potato beetle, *Leptinotarsa decemlineata* Say (Coleoptera: Chrysomelidae) [35]. The concurrent discovery of putative aggregation pheromones in *Aphthona* [21], as well as the later identification of aggregation pheromones in two other genera used in weed biocontrol [15,18], could prove valuable in developing a foundation for understanding the complex nature of behaviors mediated by aggregation pheromones in the Chrysomelidae.

## 5. Plant-Produced Semiochemicals

Volatile organic compounds (VOCs) are released from plants and may act as semiochemicals, for instance providing information on the suitability of a plant as a host [7,8]. Plant tissue damaged by herbivory results in a profile of VOCs, characteristic of the plant species and the nature of the herbivory [36,37,38], known as herbivore induced plant volatiles (HIPVs). The HIPVs include three broad categories of compounds; terpenoids, aromatic compounds and the green leaf volatiles (GLVs). The HIPVs can transmit substantial information on the nature of the herbivore attack and this information may be perceived by numerous organisms associated with the injured plant [37,38]. Although they are part of plant defense systems, they may also be used by herbivores to call in mates in conjunction with sex pheromones, and to locate host plants in conjunction with aggregation pheromones [39].

In the *Tamarix-Diorhabda* system, adult males locate suitable host plants, feed, and emit the pheromone blend while inflicting feeding damage which dramatically elevates GLVs, part of the suite of volatiles *Tamarix* produces in response to herbivory [16,40]. A combination of the male-produced pheromone blend and GLVs attracts conspecific adults to alight, feed, mate and oviposit [15,16] bringing about high densities of larvae and defoliation of shrubs in as little as two weeks [41]. Multiple defoliations can, in some cases, kill plants [42,43]. While GLVs are highly attractive to starved beetles, such as found after overwintering (e.g., Figure 3) or just after adult emergence, in most cases GLVs are less attractive to beetles than the pheromone blend [23]. The level of response in relation to pheromone response depends on the ecological setting and the physiological status of the adults [40,44]. In addition, there are at least 15 VOCs from *Tamarix* that are antennally active, indicating the complexity of semiochemicals utilized by *D. carinulata* [40] and the possibility of discovering additional behaviorally active VOCs. 

In a study of *Galerucella* pheromone biology it was shown that traps baited with six antennally active GLVs failed to increase *Galerucella* capture numbers [19]. It was clear that GLV baited traps were not attractive in the early summer, however tests were not made at other times during the season when the ecological and physiological context may have been favorable to GLV-mediated attraction. In a laboratory setting host plant odors from mechanically damaged *L. salicaria*, combined with pheromone, were more attractive to male *G. pusilla* than was the pheromone alone [45], while females showed no increased attraction to pheromone in the presence of host plant odors. The above results indicate a complex relationship between plant-produced semiochemicals and the *Galerucella* pheromone in mediating behavior of these agents. 

As reproductive *D. carinulata* move through the landscape they form swarms of adults that locate and aggregate on plants that had been previously unoccupied by conspecifics. We have observed beetles bypassing colonized trees that have an abundance of green foliage. This led us to hypothesize a chemical signature indicating *D. carinulata* density, emitted perhaps by eggs or feeding larvae, or by the *Tamarix* plants following feeding damage. Such a chemical signature would enable reproductive beetles to assess the density of conspecifics and avoid trees likely to be defoliated in the near future which would endanger their offspring, a system similar to what has been described in species of bark beetles (Coleoptera: Curculionidae) [46]. 

**Figure 4:**
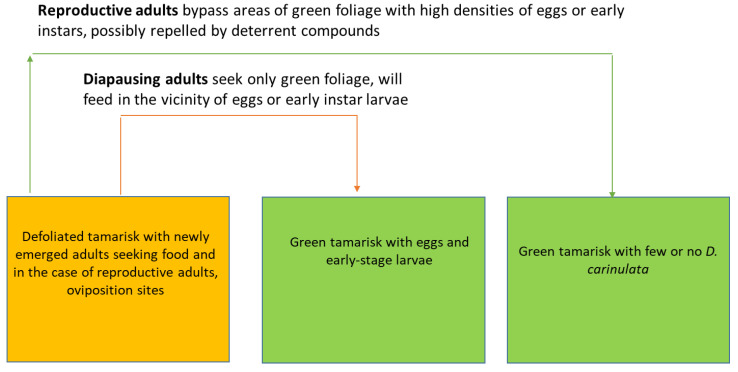
Schematic representation of dispersing *D. carinulata* adults moving out of defoliated areas and seeking green tamarisk for feeding and oviposition (reproductive state) or simply feeding (diapause destined state).

An HPIV with repellent properties was isolated from *Tamarix* that had been damaged by feeding *D. carinulata* [17]. The compound, 4-oxo-(*E*)-2-hexenal, was repellent to reproductive *D. carinulata* but did not repel diapause-destined (i.e., non-reproductive) adult beetles. This matched the hypothesized behavioral response if repellency served primarily to prevent oviposition on previously occupied shrubs and starvation in the subsequent generation of larvae (Figure 4). Diapause-destined adults, on the other hand, need only to feed a few days to achieve sufficient metabolic reserves prior to descent into the leaf litter for the winter [44]. While adult *D. carinulata* may use the compound to gauge the density of larval conspecifics, it likely serves the plant as a defense against generalist feeders since it is known to be neurotoxic to some insect species, while it has low toxicity, even in high dosages, to co-evolved *D. carinulata* [17].

## 6. Using Semiochemicals and the Principles of Chemical Ecology to Enhance Weed Biocontrol

The examples of semiochemical use in weed biocontrol illustrate the potential of using chemical ecology at multiple points in weed biocontrol implementation. Implementation can be divided into three steps; establishing agents, monitoring agents, and manipulating agent populations after they have become established. 

### 6.1. Enhancing Establishment: Importance of Pheromones during Rearing and Releases

Production of aggregation pheromone is presumed to be essential to colonization success, including mate finding and host plant interaction, of both *Diorhabda* spp. and *Galerucella* spp. so maximizing aggregation pheromone production should enhance field establishment. When *G. calmariensis* were fed alternative hosts instead of purple loosestrife, pheromone production was severely inhibited [19]. While survivability of the agent on alternative hosts also decreased, this scenario highlights the importance of nutrition for pheromone production. The propagation of healthy populations of targeted invasive weeds in a greenhouse, as food for an insect colony, can be extremely time intensive and difficult. Use of alternative hosts or artificial diets should only be considered if they do not impact pheromone emissions. Even after switching back from nontarget hosts to the target weed it took *G. calmariensis* males several days to recover pheromone emissions to pre-treatment levels [19]. When insects that have been raised under sub-optimal conditions are released in the field, they will be unable to immediately form aggregations, which could make them susceptible to density dependent fitness factors, such as Allee effects [47,48]. The host diet and periods of starvation become especially important to species that specifically sequester or acquire host plant compounds and use them as pheromones or as the precursors to pheromones [26,39]. If the wrong host plant is used or if host plants are in poor condition, the insects may not have the necessary precursors to produce the pheromone, which could compromise establishment. 

When agents are processed for shipping and field releases, they are commonly packaged in high densities in opaque containers, with or without host foliage, and remain in cool dark conditions for several days (Figure 2B, [49]). This can result in starvation and other forms of stress such as disruption of photoperiodic cues needed to maintain insects in a reproductive state necessary for oviposition and production of the aggregation pheromone blend [44]. Experiments conducted on *D. carinulata* showed that the typical process of packaging insects for release in the field compromised the males’ ability to produce the aggregation pheromone [50]. Following removal from conditions simulating shipping, it took 24 h of feeding on host plants before males achieved a pheromone release rate equivalent to those under control conditions. In a test of small releases of *D. carinulata,* with and without synthetic aggregation pheromone, field-released insects disappeared rapidly from releases sites, while the addition of continuous release formulations of the pheromone resulted in retention and reproduction of insects at experimental release sites [50]. The addition of pheromone dispensers to field release sites was used to overcome the detrimental effects of shipping on pheromone emissions, resulting in better establishment of the agent.

### 6.2. Sentinel Trapping and Population Monitoring 

Once an agent has been reared and released into the field, the next critical phase of a weed biological control program is monitoring for agent establishment and population growth. Post release monitoring has been incorporated into the code of best practices for weed biocontrol [51] yet it remains difficult to fulfill, particularly on a landscape scale. 

Under some circumstances, insect damage can be used as a proxy for the establishment of the agent and population density, especially when the damage is specific to the agent and the target weed has few other insect herbivores [52,53]. However, many agents, such as stem borers, damage plants in ways not readily visible or in ways similar to other insect damage. Periodic feeding from a generalist herbivore may prevent the proper assessment of the damage from a biocontrol agent when it has been newly released and is at very low population densities. The development of low-cost pheromone traps would be advantageous to most biocontrol programs, minimizing material and labor cost, while also generating extensive and efficient survey data, even when the agent is at a low density.

- The use of the sex attractants allowed researchers to determine that five pairs of the gorse pod moth, *Cydia succedana* were sufficient to get establishment, thus eliminated costly mass release. The discovery that so few adults were needed for establishment enabled researcher to target many more sites for release [10]. In Hawaii, the pheromone-based monitoring for *Acleris* (=*Croesia*)* zimmermani* Clarke 1978 (Lepidoptera: Tortricidae) and *Schreckensteinia festaliella* Hübner (Lepidoptera: Schreckensteiniidae) resulted in better evaluation of the *Rubus* spp. [11] biocontrol programs by providing cost-effective presence/absence data, as well as data on density, phenology, host plant synchrony, and dispersal rates [27].
- Aggregation-causing semiochemicals, especially the aggregation pheromone, were successfully deployed in the field to monitor for the presence of the biocontrol agent *D. carinulata*. The pheromone was deployed in conjunction with passive, yellow sticky card traps and resulted in detecting the early establishment of *D. carinulata* at six locations in the southwestern United States, allowing land managers to plan accordingly to incorporate the biological control program into their broader land management strategy [T. Dudley, unpublished data]. The deployment of pheromone-baited traps for detection of *D. carinulata* also provides an example of how semiochemical baits can be effectively used to detect low-level presence during the initial range expansion of a newly established agent. In the case of *Tamarix* biocontrol *D. carinulata*-induced defoliation was readily apparent and detectable using remote sensing (e.g., [53]) but the initial colonization events, presumably driven by dispersal from areas with more dense populations, were extremely difficult to detect without semiochemical baits [16]. This is due to the vast areas covered by *Tamarix*, and the patchy distribution of *Diorhabda* spp. during initial colonization, which means that sweep sampling using insect nets can easily miss early colonizing populations.

### 6.3. Directing Activity: Manipulating Population Density with Attractants & Deterrents

New research has demonstrated that for *D. carinulata,* field deployment of lures containing the aggregation pheromone (but with no sticky traps as above) resulted in increased densities of the agent in targeted *Tamarix* stands [23,54]. The deployment of the aggregation pheromone effectively allowed researchers to dictate where the beetles aggregated and fed. This resulted in increased damage and dieback to the targeted plants, especially in areas where the agent was present in low densities and where baseline damage was minimal. For *Tamarix*, two years of defoliation commonly results in a 25–33% reduction in plant canopy. However, when *D. carinulata* was directed to target plants with the aggregation pheromone, there was a 73–79% reduction in canopy size after two years of defoliation [23,54].

Like most invasive plants, those in the genus *Tamarix* have the ability to recover from or tolerate limited feeding from herbivores. Using semiochemicals to artificially increase the densities of a biocontrol agent could help to overcome tolerance levels of the target plant and result in more efficient and faster control outcomes for the biocontrol program. Providing resource managers with the means to focus the impact of a biological control agent to targeted areas could prove widely useful for invasive plant biological control. For instance, if a resource manager has a stand of *Tamarix* and *D. carinulata* present nearby, but not present in numbers to cause significant impact, the aggregation pheromone could be deployed to artificially increase *D. carinulata* densities to enhance the impact on targeted *Tamarix* stands. It has also been proposed that this strategy could be used to control new infestations of the target weed, especially where it is mixed with native plants. The selective removal of an invasive weed from a mixed species community is time-consuming and can result in significant damage to the native plants. However, directing host specific biocontrol agents to areas with the target weed would allow for minimal impact to the native plants while also conferring a competitive advantage to them over the target weed. The select removal of an invasive plant before it displaces the native one can reduce the long-term negative environmental impacts of weeds.

Repelling biocontrol agents from an area may be a resource management objective under some circumstances. Invasive plants can provide limited ecosystem services when native plant communities have been seriously degraded. Temporarily sustaining those services by limiting biocontrol impacts on strategically located stands of the target invasive plant can be beneficial while ecosystem restoration and recovery are underway [43]. This scenario is being played out in the southwestern United States, where localized defoliation of *Tamarix* can reduce its suitability as nesting habitat for an endangered bird subspecies, the southwestern willow flycatcher, *Empidonax traillii extimus* (Passeriformes: Tyrannidae). In the long-term, weed biocontrol is likely to prove beneficial in recovery of this subspecies, especially if riparian ecosystem restoration is employed as part of the recovery plan [43]. However, in the interim, interactions between *Tamarix* biocontrol and the endangered bird could be minimized by manipulation of the *Diorhabda* population through use of semiochemicals. 

**Figure 5:**
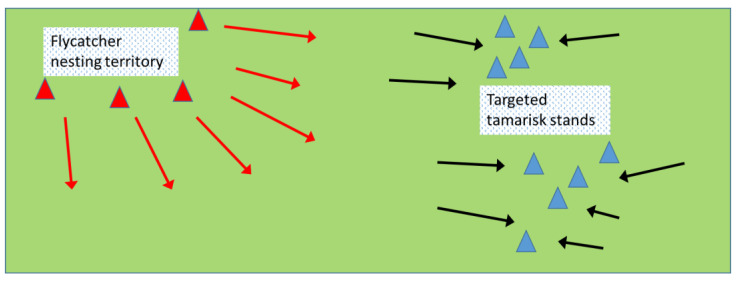
A push-pull semiochemical strategy to repel reproductive adult *D. carinulata* away from nesting territories of the endangered southwestern willow flycatcher, where tamarisk may be utilized [43] and attract them to areas where tamarisk is targeted for removal. The repellent (red triangles) is placed near nesting territories where it diminishes the number of adult beetles colonizing tamarisk shrubs [17]. The *D. carinulata* pheromone blend (blue triangles) is placed on trees targeted for removal. Beetles are attracted (black arrows) to the pheromone, resulting in elevated *D. carinulata* populations and accelerated defoliation of the shrubs [23,54]. Targeted areas with blue triangles will be defoliated early in the season while the flycatcher nesting territory will remain green until later, after birds have fledged.

Push-pull strategies involve the behavioral manipulation of insects using the integration of stimuli that act to make the protected resource unattractive or unsuitable to the insect (push) while luring them toward an attractive source (pull) [55]. It has been hypothesized that *Diorhabda* populations could be manipulated using a push-pull system that combines *D. carinulata*’s aggregation pheromone (pull) with the plant compound 4-oxo-[*E*]-2-hexenal, newly discovered to be repellent to reproductive *D. carinulata*, which would be the push [17]. Strategically placed semiochemical baits would result in directed defoliation of *Tamarix* to stands outside of flycatcher nesting territories, while deterring defoliation at flycatcher nest sites during breeding season ([17], Figure 5). The ability to manage tamarisk stands for flycatcher nesting habitat would defuse the controversy that has slowed a successful biocontrol program [56]. Repelling a biological control agent from an area occupied by the target weed runs counter to the usual desired outcomes from a biological control program, but if a repellent can be used to minimize transient direct or indirect negative interactions between a biological control agent and non-target organisms it could be very useful in an integrated weed management program.

## 7. Future Directions

The increased application of semiochemicals and chemical ecology principles to host range analysis has been proposed by Wheeler and Schaffner for the practice of weed biocontrol [8] with a primary focus on plant secondary compounds as determinants of host range and coevolved host-plant relationships. We would like to expand the Wheeler-Schaffner proposal to include post release evaluation and manipulation of weed biocontrol populations using semiochemicals. This can be accomplished through a systematic analysis of field behavior including host plant searching and mate location, as well as other host plant-herbivore interactions. By observing behavior patterns shown by agents in the field and laboratory, researchers can formulate hypotheses and define points where semiochemicals play critical roles in the biology of a biocontrol agent. Biocontrol agents and their target plants are both possible sources of semiochemicals and the interplay of chemicals from both sources could be essential for modulating biocontrol agent behavior. An increased understanding of chemical ecology would be useful in mitigating some instances of underperformance or failures in the implementation of weed biological control. In some cases, semiochemicals would be valuable tools for practitioners in optimizing classical biocontrol of invasive target plants. Additional research will also be needed to evaluate the impacts of semiochemical deployment on parasitism and predation of the biocontrol agent as higher trophic levels are also deeply impacted by semiochemicals [38,57]. It is possible that the interaction with higher trophic levels may minimize the benefits of push-pull strategies for weed biocontrol programs. 

The pathways for discovery of behaviorally active compounds have been defined, e.g., [15,18,21] and in some cases all or most of these steps have been completed and it only remains to test practical uses for identified semiochemicals in weed biocontrol, e.g., [21]. In other instances, further investigation and development are required before practical uses of semiochemicals can be discovered. For example, in the case of flea beetles, *Aphthona* spp, used against leafy spurge, the agents are considered effective in many habitats, but some areas where the agents establish never see sufficient control [58]. Putative aggregation pheromones for these species have been identified and chemical synthesis has been developed [21,59]. Behavioral activity in the field remains to be shown but should the compounds prove to be aggregation pheromones they could be deployed during new releases to help retain larger numbers of the agent at the release site since they may not be capable of emitting their own pheromone shortly after being released [54]. Monitoring is likely to be enhanced by using pheromones to track populations, and density manipulations could be conducted to achieve greater impacts from the established populations. Ultimately these additional strategies would result in significant improvements in this biocontrol program. 

In contrast to the above examples, most weed biocontrol agents have not been investigated for semiochemical involvement in behavior and host plant interactions. Other weed biocontrol agents, particularly adult beetles, are found in aggregations, making it reasonable to hypothesize semiochemical involvement in bringing and keeping them together on the host plant. Discovery and development of semiochemicals in aggregating species would begin with isolation of antennally active compounds and proceed from there and could lead to enhancement of establishment and efficacy in currently uninvestigated agents. We have noted aggregation behavior in the seed head feeding weevil *Larinus minutus* Gyllenhall (Coleoptera: Curculionidae) on diffuse knapweed (*Centaurea diffusa* Lam.) and the stem boring weevil *Mecinus janthiniformis* Tosevski & Caldara (Coleoptera: Curculionidae) on Dalmatian toadflax (*Linaria dalmatica* (L.) Mill.) and there are undoubtedly many more examples of aggregation among biocontrol agents. 

Sex pheromones are another class of semiochemicals that has proven useful in weed biocontrol [27]. The pathways for isolation and identification of sex pheromones are well defined, given the long-standing use of pheromones in monitoring and mating disruption in agricultural systems [60]. Pheromone-based monitoring systems could be utilized to monitor low-density populations as well as to glean information necessary to evaluate synchrony of a newly introduced weed biocontrol agent with host plant phenology [9,10,11]. Phenological asynchrony, determined by flight time measured with pheromone-baited traps, is thought to be a factor in suboptimal biocontrol performance of the gorse pod moth with the host plant [9]. Measuring flight times of reproductive biocontrol agents in combination with plant seasonal availability could provide data to evaluate the need for agents better adapted to the introduced range. 

This report has been directed toward the use of chemical ecology in the implementation of a weed biocontrol program, distinguished from the activities associated with safety and host specificity of agents. Information on semiochemical signaling pathways can inform both sets of activities. For instance, the discovery that *Galerucella* males fail to emit aggregation pheromone when feeding on non-target suboptimal hosts [19] provides additional evidence of host specificity and safety of the loosestrife beetles. There have been several recent studies addressing differences in VOC profiles between biocontrol target weeds and native nontarget species [61,62,63,64,65]. These studies focused on the behavioral responses to the VOCs and the implications for host selection and safety. While safety was of primary concern, the discovery of semiochemicals with antennal activity in biocontrol agents could inform the implementation and management of biocontrol agents once released. For instance the established biocontrol agent for musk thistle (*Carduus nutans* L.), *Rhinocyllus conicus* Frölich, (Coleoptera: Curculionidae) is known to select native thistles in the field. Studies on differences between VOC profiles of the target and native species may lead to development of repellent semiochemicals to diminish attack on native thistles. This possibility was suggested by the authors of that study as a way to use repellent compounds to manage for endangered thistles [65]. Studies in which response of the biocontrol agent *Mogulones borraginus* F. (Coleoptera: Curculionidae) to volatile components from the target-hound’s tongue (*Cynoglossum officinale* L. Boraginaceae) were compared to those from non-target species to help define the host range of this insect [61,62]. While these studies aimed to address questions concerning safety of *M. borraginus* and their responses to semiochemicals in combination with visual cues, the findings revealed semiochemical attractants produced by *C. officinale*, as well as repellents produced by non-target species. The information and methodologies used in those studies could readily apply to implementation programs using *M. borraginis*, where attractants and repellents would be very useful. 

Once semiochemicals have been identified in weed biocontrol agents, the last barrier to incorporation into weed management programs is the availability of these compounds to biocontrol practitioners and resource managers. For example, the *Diorhabda* aggregation pheromones are not commercially available and so researchers must either synthesize the pheromone or work with chemists to synthesize it prior to initiating experiments [15,23,50,54]. In addition to pheromone availability, field release methods need to be developed prior to deployment. For the *Diorhabda* pheromones this was accomplished through a cooperative project with a private company (ISCA Technologies, Riverside, CA, USA), to incorporate semiochemicals into a commercially available flowable wax-based controlled release matrix known as SPLAT^®^. The formulation was shown to provide an acceptable release rate for the semiochemicals even after 30 days in the field [23]. While effective for research purposes, this formulation was project-specific and is not commercially available, and would not be economically feasible to produce for routine low volume use. If a critical economic threshold is exceeded, the synthesis and formulation of semiochemicals can be economically favorable and performed by private companies. However, low initial demand makes this unlikely and the lag between discovery and economic feasibility of large-scale synthesis and commercial development has been called “the valley of death” [66] for specialty semiochemicals. Strategies to overcome this, including development of partnerships with private companies and establishment of consortia to ensure semiochemical availability, should be explored to make semiochemicals available to end users. 

## 8. Conclusions

These examples are meant to stimulate discussion and research on the use of semiochemicals as tools for biological control practitioners to increase efficacy of weed biocontrol. When implementing a biological control program, the potential benefits of the insect pheromones and other semiochemicals should be considered, as they can be a critical factor in successful establishment, monitoring, and impact of the program. The integration of chemical ecology and weed biocontrol offers fertile research opportunities that can enhance the services provided by biological control programs.

## Figures and Tables

**Table 1:** Weed biological control agents with identified semiochemicals. Three uses for identified semiochemicals are enhancing agent establishment (EE), sentinel trapping and monitoring (STM) and directing activity (DA). These categories are described in detail in the section on enhancing weed biocontrol. Herbivore-induced plant volatiles are abbreviated HIPVs.

Scientific Name	Order: Family	Agent Common Name and Host Plant	Semiochemical Type and Uses	Reference
*Agonopterix ulicitella*	Lepidoptera: Oecophoridae	gorse shoot moth/*Ulex europaeus*	sex attractant/STM	Suckling et al. [9]
*Cydia succedana*	Lepidoptera: Tortricidae	gorse pod moth/*Ulex europaeus*	sex attractant/STM	Suckling et al. [10]
*Acleris (=Croesia) zimmermani*	Lepidoptera: Tortricidae	none/*Rubus* spp.	sex attractant/STM	Suckling et al. [11]
*Schreckensteinia festaliella*	Lepidoptera: Schreckensteiniidae	blackberry skeletonizer/*Rubus* spp.	sex attractant/STM	Suckling et al. [11]
*Tyta luctuosa*	Lepidoptera: Noctuidae	field bindweed moth/*Convolvulus arvensis*	sex pheromone/STM	Cao et al. [12]
*Agapeta zoegana*	Lepidoptera: Tortricidae	sulphur knapweed moth/*Centaurea* spp.	sex pheromone/STM	Tóth et al. [13]
*Cactoblastis cactorum*	Lepidoptera: Pyralidae	cactus moth/*Opuntia* spp.	sex pheromone/STM	Heath et al. [14]
*Diorhabda carinulata*	Coleoptera: Chrysomelidae	northern tamarisk beetle/*Tamarix* spp.	aggregation pheromone blend/EE, STM, DA	Cossé et al. [15]
*D. carinulata*	Coleoptera: Chrysomelidae	northern tamarisk beetle/*Tamarix* spp.	*Tamarix* HIPVs attractants/ STM	Cossé et al. [16]
*D. carinulata*	Coleoptera: Chrysomelidae	northern tamarisk beetle/*Tamarix* spp.	*Tamarix* HIPV deterrent/ DA	Gaffke et al., [17]
*Galerucella calmariensis* and *G. pusilla*	Coleoptera: Chrysomelidae	black-margined and golden loosestrife beetles/*Lythrum salicaria*	aggregation pheromone/STM	Bartelt et al. [18,19]
